# Fertilization, embryo culture, and clinical results using low lactate embryo culture medium for pre‐culture, insemination, and beyond

**DOI:** 10.1002/rmb2.12458

**Published:** 2022-04-06

**Authors:** Masato Kobanawa

**Affiliations:** ^1^ Kobanawa Clinic Omitama‐shi Japan

**Keywords:** embryo metabolism, glycolysis, lactate, oxidative phosphorylation, pyruvate

## Abstract

**Purpose:**

We focused on the metabolism of oocytes in pre‐culture and insemination and compared these results between our existing fertilization medium, GEMS Fertilisation Medium (GEMS group) (Merck BioPharma) and Continuous Single Culture Medium—NX Complete (CSCM‐NXC group) (FUJIFILM Irvine Scientific).

**Methods:**

Patients under 42 years of age were received controlled ovarian stimulation and oocytes were retrieved. Those were pre‐cultured and fertilized with either GEMS fertilization medium or CSCM‐NXC. After fertilization was confirmed, embryos were cultured using CSCM‐NXC in both groups. The embryos were cryopreserved at blastocyst stage (3BB or more, Gardner classification) and then transferred in HRT cycles.

**Results:**

The fertilization rate of both groups was the same, but the 3PN rate was significantly lower in the CSCM‐NXC group. In terms of embryo culture results, the CSCM‐NXC group had a significantly higher rate of good quality blastocysts, high‐grade embryos, and embryos with a high degree of expansion.

**Conclusions:**

The use of CSCM‐NXC, a low lactate embryo culture medium, from pre‐culture and for insemination, increases the energy metabolic efficiency of oocytes and cumulus cells, making it possible to supply sufficient energy ATP for fertilization and early division, which is thought to promote good embryonic development.

## Graphical abstract

The use of CSCM‐NXC, a low lactate embryo culture medium, from pre‐culture and for insemination promotes good embryonic development.

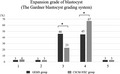

## 1 INTRODUCTION

The concept of "back to nature" led to the development of embryo culture media based on human oviduct fluid, followed by blastocyst transfer in the 1990s,^1^ and the development of a sequential medium by Gardner et al.^2^ in 1995. In contrast to this concept, the model of "Let the embryo choose" is based on constituent optimization using a statistical approach to determine the necessary components of the embryo culture medium.

In 2002, based on KSOM, Biggers et al.^3^ developed a single step medium that can be used for both early and late culture in a single medium. Single step medium was developed by Biggers et al.^4^ in 2002.

Continuous single culture medium—NX complete, the modern embryo culture medium that we focused on, is based on KSOM, and is an embryo culture medium created based on the concept of "Let the embryo choose." It is considered to be an innovative embryo culture medium that succeeds in increasing the metabolic efficiency of the embryo by greatly reducing the lactate concentration refined by the constituent optimization method.

The embryonic metabolism at the early oocyte stage, up to the third day of culture, is a period when the energy production mechanism essential for cell division is changed from mitochondria to cytoplasmic glucose metabolism, and the glycolytic system is transformed into the main pathway of energy production during the development to the blastocyst stage embryo^5^, ^6^, ^7^, ^8^

It is expected that the use of CSCM‐NXC, which is an embryo culture medium with a lower lactate concentration, promotes oxidative phosphorylation by the citric acid circuit due to the high uptake of pyruvate in the early culture,^8^ and that the supply of the cofactor NAD⁺ by the reduction in pyruvate to lactate is enhanced in the late culture due to the low amount of the metabolite lactate in the culture medium. The supply of NAD⁺ is enhanced, and energy metabolism in the glycolytic system, which uses NAD⁺ to metabolize glucose, is thought to be promoted.

In addition, the metabolic patterns of the oocyte, fertilized zygote, and early embryo are said to be similar.^9^ The oocyte uses pyruvate to metabolize energy by oxidative phosphorylation through the TCA circuit and electron‐transfer system, which serves as an important energy source during meiotic maturation.^10^, ^11^

Normally, pyruvate is transferred from the cumulus cells to the oocyte via the gap junction, but pyruvate in the culture medium is directly utilized by the oocyte.^9^

Therefore, we hypothesized that CSCM‐NXC, a low lactate culture medium, could have a positive effect on culture and clinical outcomes by increasing the energy metabolism efficiency of oocytes during pre‐culture and during insemination.

## 2 MATERIALS AND METHODS

GEMS Fertilisation Medium (GEMS) (Merck BioPharma) and Continuous Single Culture Medium—NX Complete (CSCM‐NXC) (FUJIFILM Irvine Scientific) were used as culture media for comparison. In GEMS, concentrations of lactate and glucose are about 3.0 and 5.0 mM. Those in CSCM‐NXC are 1.0 and 0.5 mM. This study was performed on patients who gave informed consent.

There was no significant difference in patient background (Table 1). The patient population consisted of 123 cases, 123 cycles, who were 36 ± 5.0 years of age and underwent IVF between January 2020 and July 2021. All patients underwent IVF between January 2020 and July 2021 participate in this study.

**TABLE 1:** Patient background of GEMS group and CSCM‐NXC group

	GEMS group	CSCM‐NXC group	*p* Value
Year	36 ± 5	36 ± 5	0.93
BMI (kg/m²)	23 ± 4	23 ± 5	0.83
AMH (ng/ml)	3.93 ± 3.17	3.64 ± 3.29	0.48
AFC	10 ± 6	9 ± 5	0.96
COS days	13 ± 2	13 ± 2	0.43
Dose r‐FSH + hMG (IU)	3079 ± 969	2992 ± 896	0.70
E₂ (pg/ml))	33.7 ± 17.1	36.8 ± 19.1	0.21
LH (mIU/ml)	6.5 ± 3.2	6.3 ± 3.1	0.78
FSH (mIU/ml)	7.4 ± 2.5	7.6 ± 3.5	0.78

Note

Data are presented as mean ± standard deviation of the mean. Patient background of GEMS group and CSCM‐NXC group was compared by Mann–Whitney's U test, and a significant difference was judged to exist when *p *< 0.05.

Control ovarian stimulation (PPOS, Antagonist or Short method) was performed and recombinant hCG was administered when the primary follicle reached 18–20 mm and oocyte retrieval was performed 34–36 h later. After oocytes retrieval, identified cumulus‐oocyte complexes (COCs) collected from follicular fluid were washed well with Multipurpose Handling Medium Complete (MHM‐C) (FUJIFILM Irvine Scientific). 67 cases from January‐September 2020 had their.

Cumulus‐oocyte complexes transferred to GEMS Fertilisation Medium (Merck BioPharma) and pre‐cultured for approximately 2 h (GEMS group). 56 cases from January to July 2021 had their COCs transferred to Continuous Single Culture Medium—NX Complete (FUJIFILM Irvine Scientific) for pre‐culture (CSCM‐NXC group). The culture was performed under 37.0°C, 6.0% CO₂, and 5.0% O₂ using a water‐jacketed personal multi‐gas incubator (APM‐30D) (Astec). On the day of oocyte retrieval, the original semen was washed by monolayer density gradient centrifugation, and the washed sperm were swum up in a smart station (SS‐250) (Astec). A Falcon organ culture dish (Falcon 353037) (Corning) was used, and 1 ml of GEMS Fertilisation Medium (Merck BioPharma) or CSCM‐NXC (FUJIFILM Irvine Scientific) was used as the insemination medium. Motile sperm was added to achieve a motile sperm concentration of 10 × 10^4^/ml, along with 1–5 pre‐cultured oocytes for insemination. After 19 h of insemination, we wash the cumulus cells and sperm and confirm fertilization. Fertilization was confirmed using an inverted microscope (OLYMPUS IX73) (Olympus Corporation) and an Intebio Station (AIM‐130) (Astec) as a clean bench. The embryos were cultured using Nipro ART CULTURE DISH 12 or 24 (NIPRO), and all embryos were cultured individually using 50 μl CSCM‐NXC (FUJIFILM Irvine Scientific) in 1 well. The embryos were observed on Day3/Day5/Day6.

In the beginning of the study, the GEMS group embryos were cultured in Continuous Single Culture Medium ‐ Complete (CSCM‐C) (FUJIFILM Irvine Scientific) and CSCM‐NXC (FUJIFILM Irvine Scientific) because a sibling study was performed for several months at the beginning of using CSCM‐NXC (FUJIFILM Irvine Scientific) experimentally. Therefore, the number of 2PN zygotes cultured in CSCM‐C (FUJIFILM Irvine Scientific) were excluded in the GEMS group results.

The early embryos on Day 3 were judged to be good quality embryos if they were 7 cells G2 or higher, and the blastocysts were judged to be good quality if they were 3BB or higher. The embryo freezing solution was Kitazato Vitrification media (KITAZATO corporation), and the device was CRYO‐TOP (KITAZATO corporation).

We compared the fertilization rate as the percentage of normal 2 pronuclei (2PN) and 3 pronuclei (3PN) rate. We also compared the percentage of 2 pronuclei, 1 pronucleus, 3 pronuclei, and no pronuclei, excluding MⅠ, MⅡ, 2 cells, and degeneration oocytes at the time of day 1 observation. The denominator of the normal fertilization rate and 3PN fertilization rate were the number of inseminated COCs.

As culture results, Day 3 good quality embryo rate (Veeck classification Grade 2 or higher), blastocyst rate (early blastocyst or higher), and good quality blastocyst rate (Gardner classification 3BB or higher) were determined and compared, respectively. The denominator of the good quality embryo rate was the number of 2PN zygotes cultured in CSCM‐NXC (FUJIFILM Irvine Scientific). The denominator of the blastocyst rate and good quality blastocyst rate were the number of 2PN zygotes which were extended in culture to day 5 or day 6.

Furthermore, we utilized blastocyst quality scores which is blastocyst scoring system developed by Khurram et al.^12^ This scoring system can change Gardner evaluation to the value. And the average value on both medium were compared.

The clinical outcomes included 89 frozen‐thawed embryo transfers in hormone replacement cycles (HRT) performed between March 2020 and August 2021. The clinical outcomes of embryos cultured in CSCM‐NXC (FUJIFILM Irvine Scientific) after GEMS (Merck BioPharma) pre‐culture and insemination and embryos cultured in CSCM‐NXC (FUJIFILM Irvine Scientific) after CSCM‐NXC (FUJIFILM Irvine Scientific) pre‐culture and insemination were compared in terms of clinical pregnancy rate and miscarriage rate. In this study, clinical pregnancy was defined as a case in which the fetal sac was confirmed by transvaginal ultrasonography among 4–5 weeks of gestation determined from the day of embryo transfer, and miscarriage was defined as a case in which the fetal sac disappeared after confirmation.

Mann–Whitney U test was used for patient background, and a significant difference was judged to exist when *p *< 0.05. (Table 1). The chi‐square test was used for fertilization results, culture results, and clinical results, and a significant difference was judged to exist when *p *< 0.05. The *t*‐test was used for the comparison of the average value of blastocyst quality scores on both medium.

## 3 RESULTS

Fertilization results, culture results, and clinical results were compared between the group that was pre‐cultured and inseminated with GEMS Fertilisation Medium and subsequently cultured with CSCM‐NXC (FUJIFILM Irvine Scientific) and the group that was pre‐cultured, inseminated, and cultured with CSCM‐NXC (CSCM‐NXC group).

**FIGURE 1:**
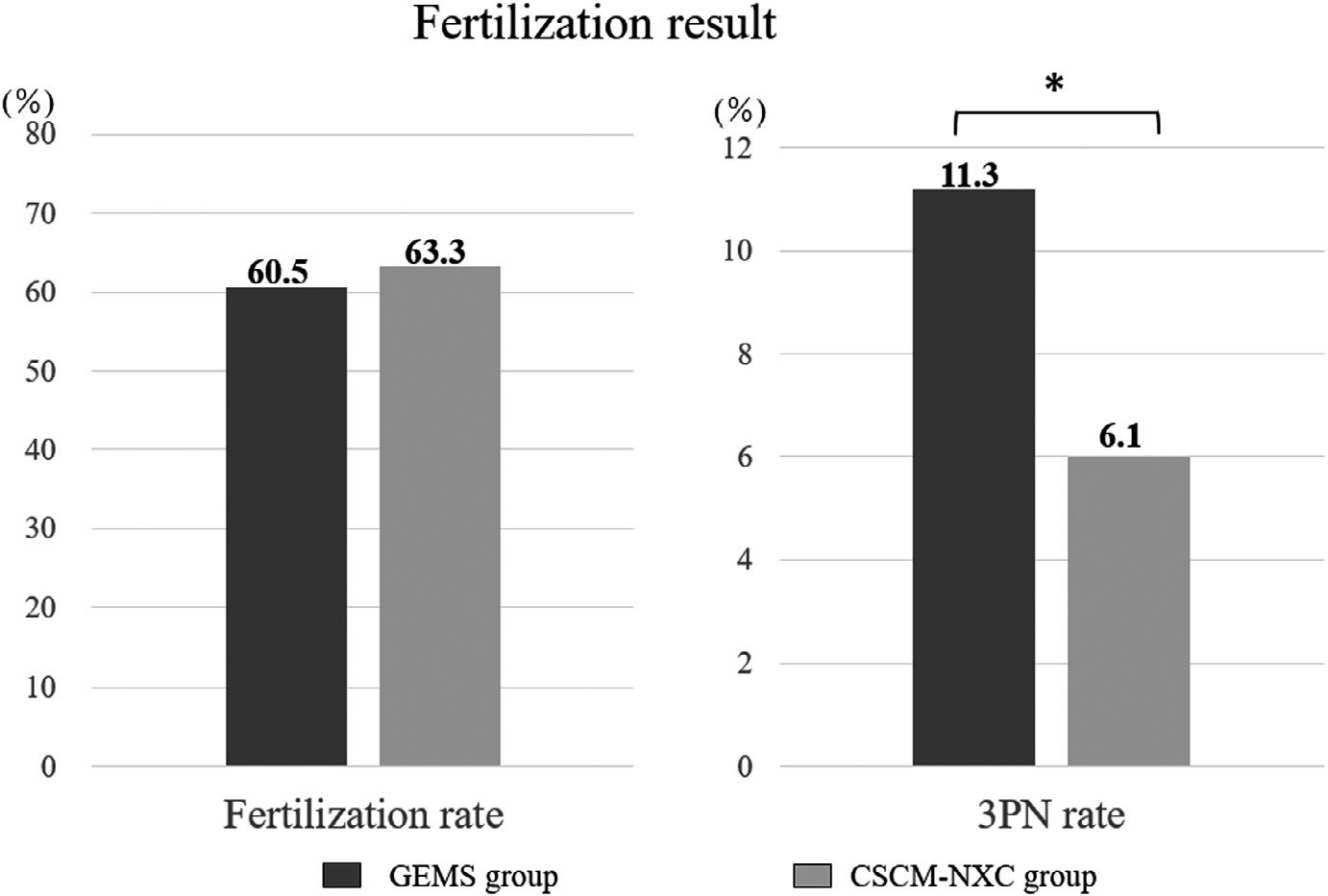
Fertilization rate was determined and compared. The black graph shows GEMS group and the gray graph shows CSCM‐NXC group

Fertilization results between the GEMS group and the CSCM‐NXC group showed that the normal fertilization rate (2PN rate) was 60.5% (505/835) and 63.3% (418/660), respectively, and the 3PN rate was 11.3% (94/835) and 6.1% (40/660), respectively. Although there was no significant difference in the normal fertilization rate, the 3PN rate was statistically significantly lower in the CSCM‐NXC group. (Figure 1).

**FIGURE 2:**
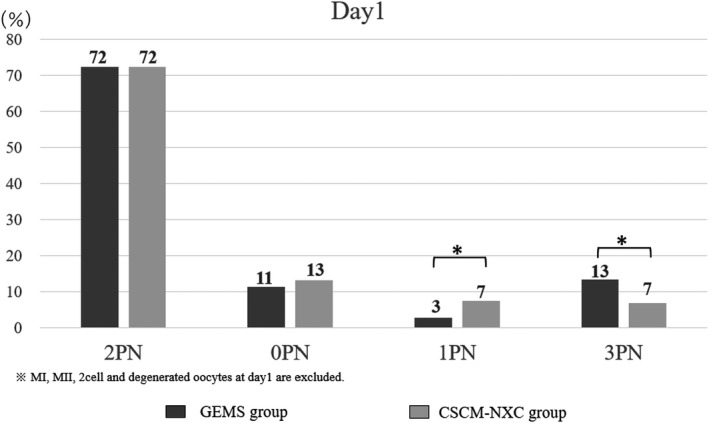
Embryonic status at day 1 was determined and compared. The black graph shows GEMS group and the gray graph shows CSCM‐NXC group

The embryonic status at day 1 for the GEMS group was 72%, 11%, 3%, and 13% for 2PN, 0PN, 1PN, and 3PN, respectively. The embryonic status at day 1 for the CSCM‐NXC group was 72%, 13%, 7%, and 7% for 2PN, 0PN, 1PN, and 3PN, respectively, with statistically significantly more 1PN as well as statistically significantly less 3PN in the CSCM‐NXC group. (Figure 2).

**FIGURE 3:**
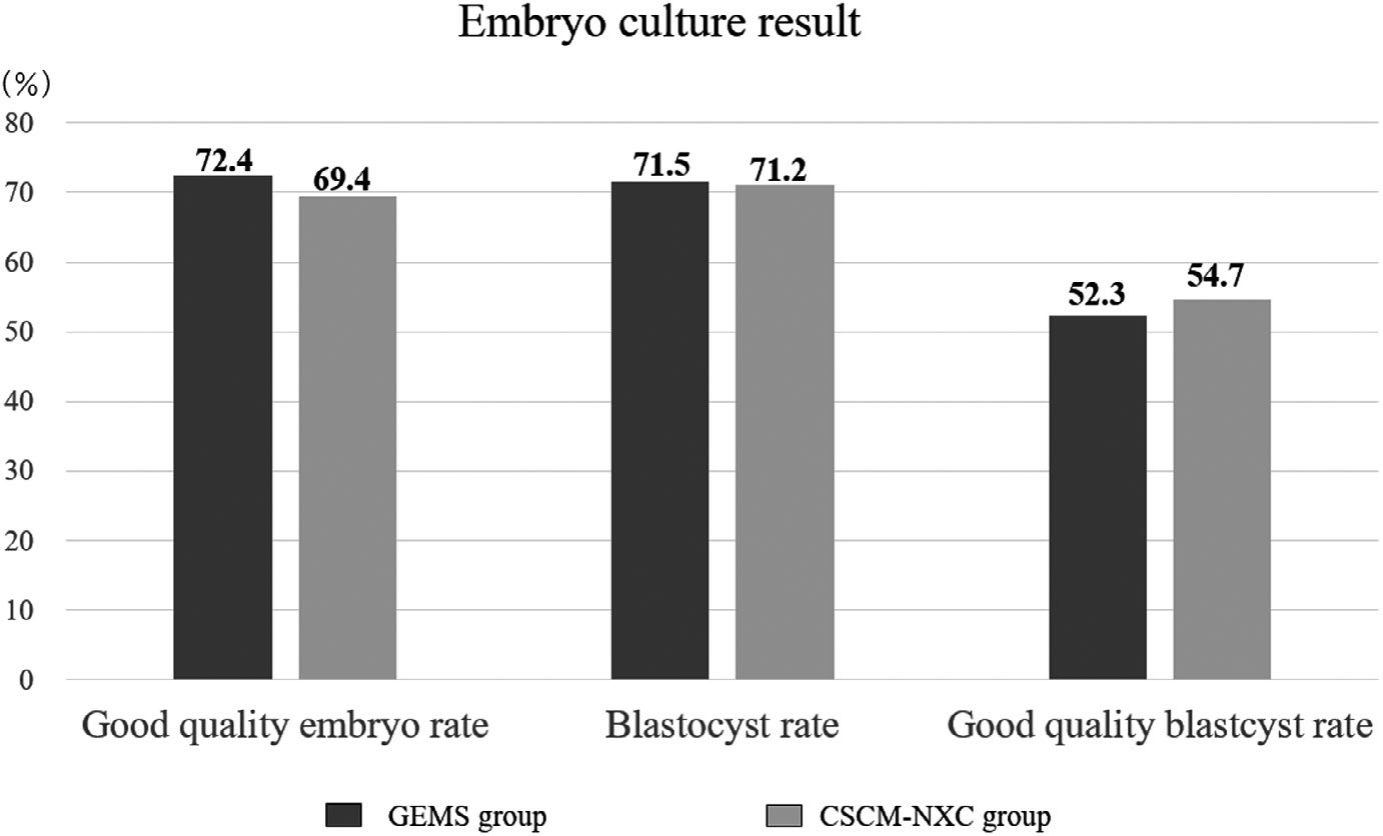
Good quality embryo rate, blastocyst rate, and good quality blastocyst rate determined and compared. The black graph shows GEMS group and the gray graph shows CSCM‐NXC group

As for the culture results, the good quality embryo rate was 72.4% (189/261) in the GEMS group and 69.4% (290/418) in the CSCM‐NXC group, the blastocyst rate was 71.5% (186/260) and 71.2% (297/417), and the good quality blastocyst rate was 52.3% (136/260) and 54.7% (228/417), respectively. There was no significant difference between them. (Figure 3).

**FIGURE 4:**
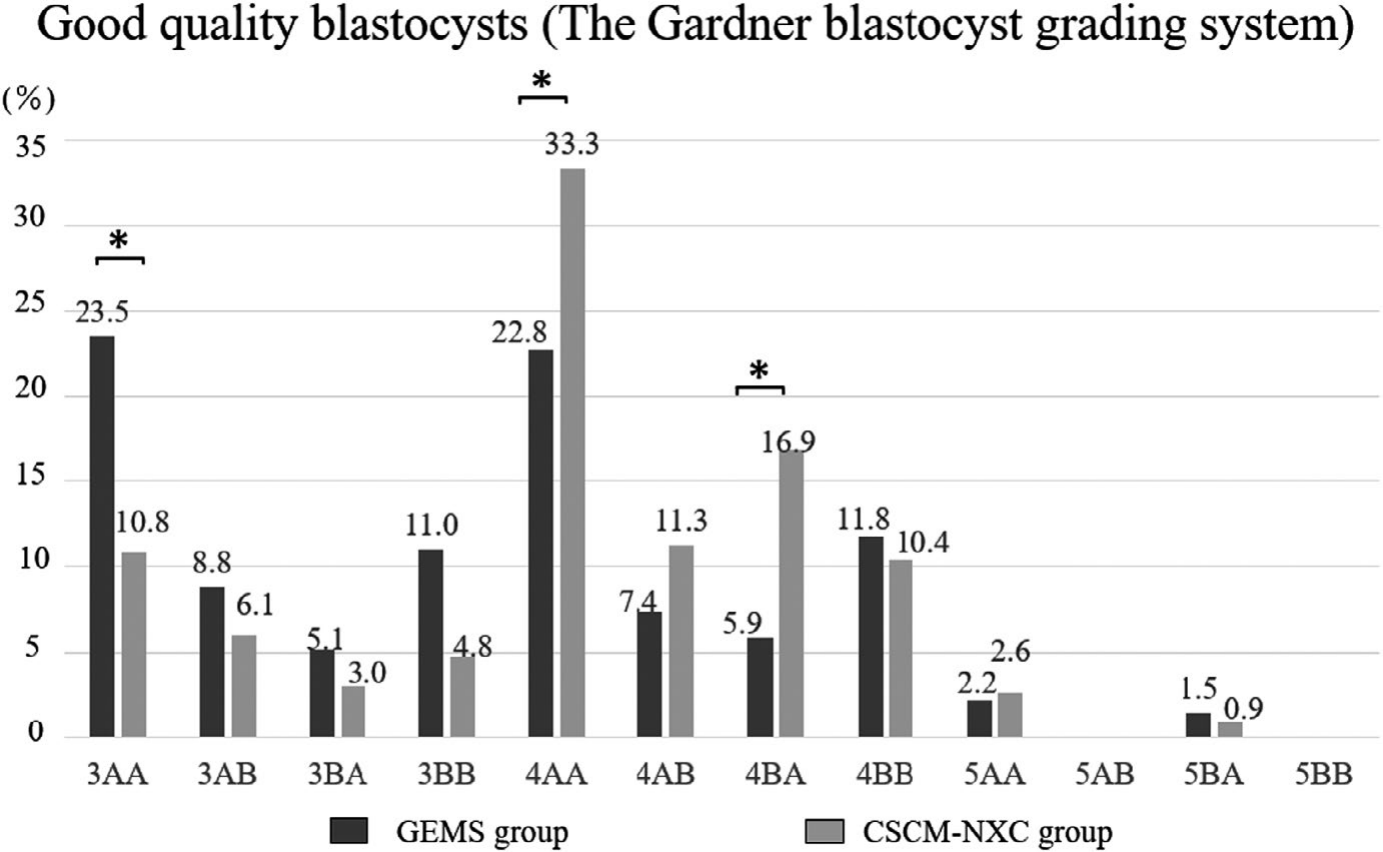
Grade of good quality blastocysts was determined and compared. The black graph shows GEMS group and the gray graph shows CSCM‐NXC group

In the comparison of good quality blastocysts by grade, 3AA was statistically significantly more common in the GEMS group at 23.5% (10.8% in the CSCM‐NXC group), while 4AA showed a statistically significant improvement in the CSCM‐NXC group at 33.3% (22.8% in the GEMS group) and the occurrence of 4BA was statistically significant in the CSCM‐NXC group at 16.9% (5.9% in the GEMS group). The CSCM‐NXC group had a significantly higher rate of blastocysts of higher quality grade. (Figure 4).

**FIGURE 5:**
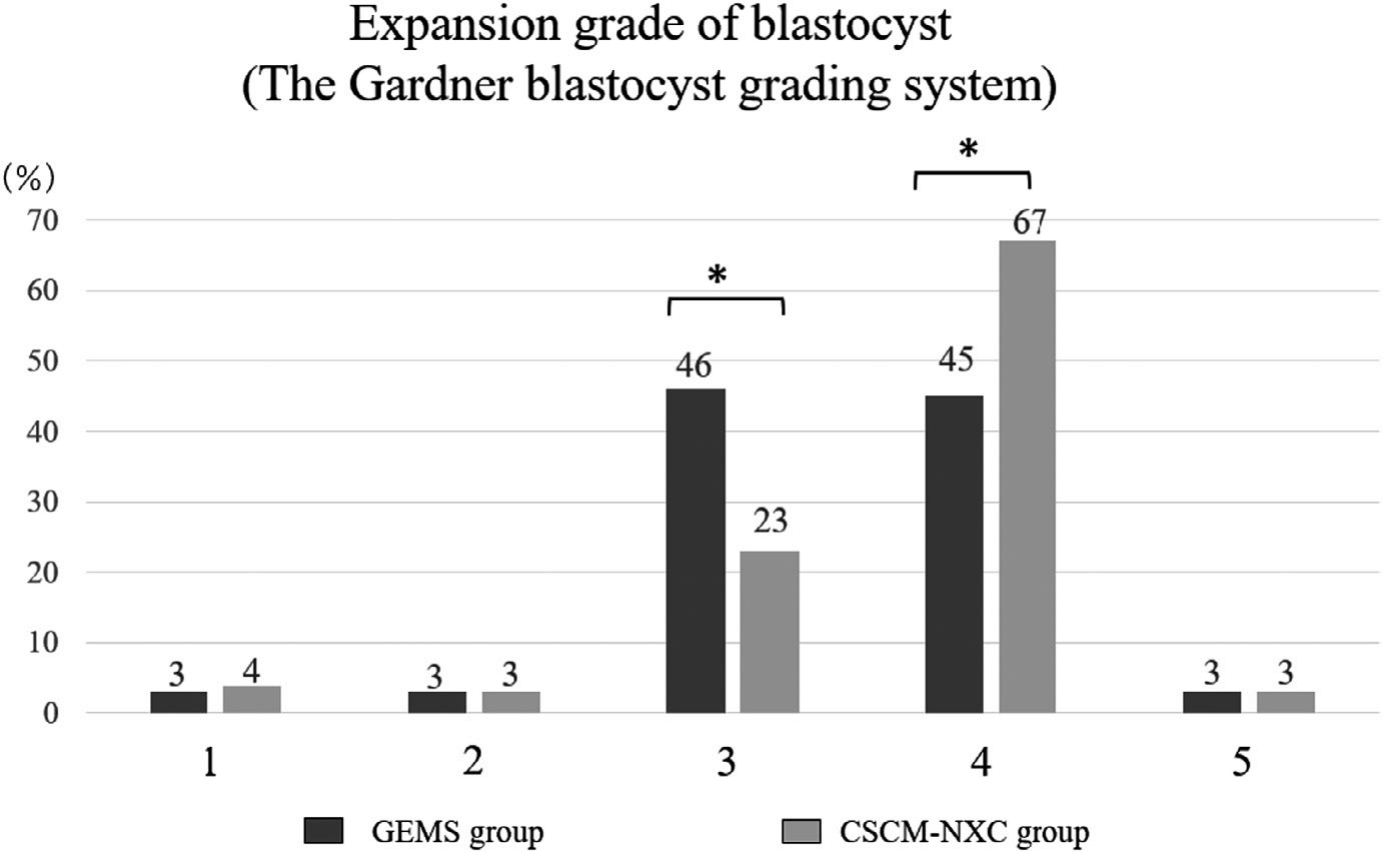
Expansion grade of blastocyst was determined and compared. The black graph shows GEMS group and the gray graph shows CSCM‐NXC group

In the comparison of blastocyst expansion grades, 46% of the blastocysts in the GEMS group and 23% in the CSCM‐NXC group were classified as blastocysts, which is significantly lower in the CSCM‐NXC group. (Figure 5) However, blastocyst expansion grade 4, expanded blastocyst stage, was 45% in GEMS and 67% in CSCM‐NXC. Thus, the increase in blastocyst rate with a higher degree of expansion is statistically significant in the CSCM‐NXC group.

**FIGURE 6:**
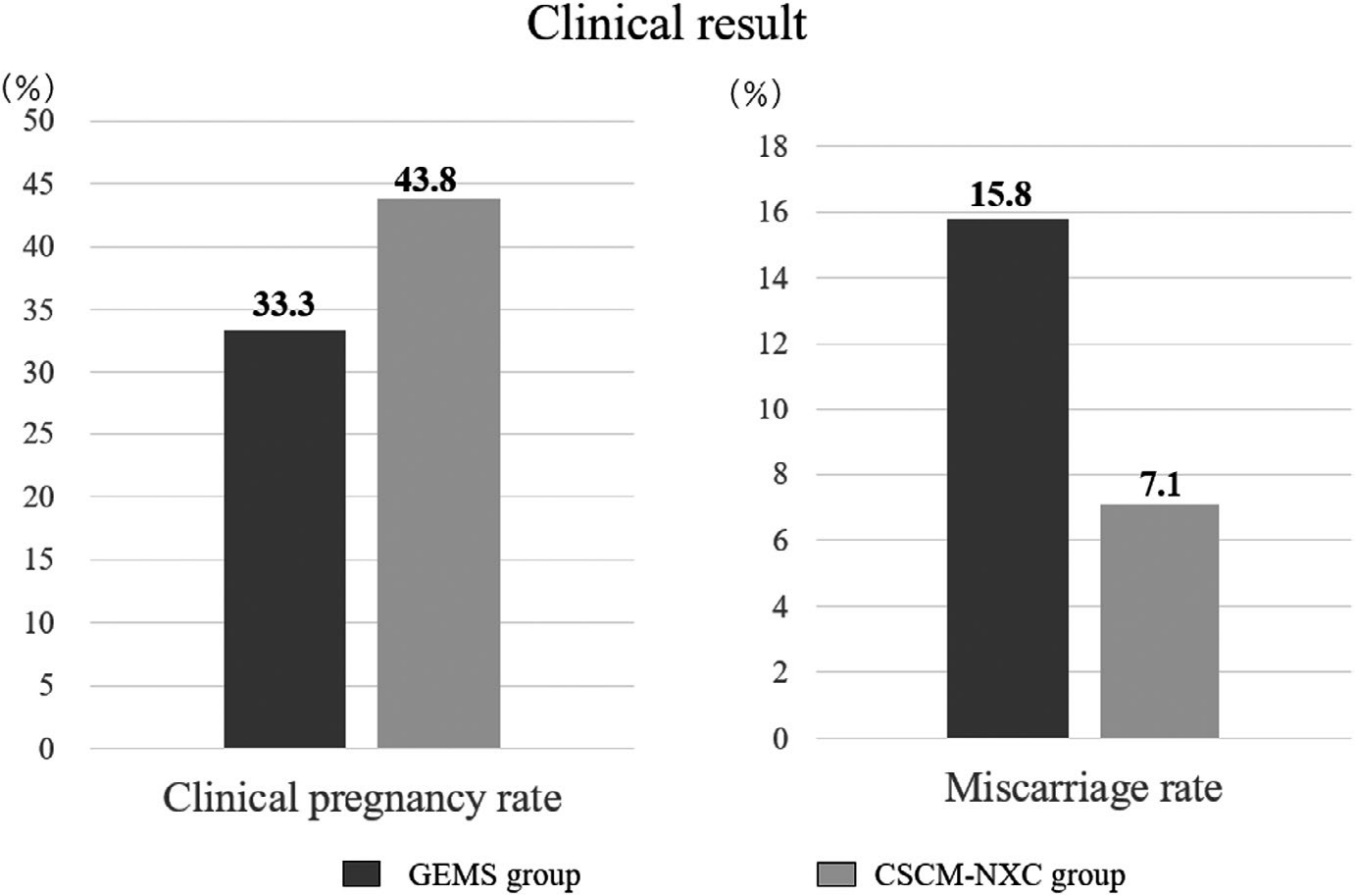
Clinical pregnancy rate and miscarriage rate were determined and compared. The black graph shows GEMS group and the gray graph shows CSCM‐NXC group

The clinical results of the embryos in both groups showed that the pregnancy rate was 33.3% (19/57) and 43.8% (14/32), respectively. The miscarriage rate was 15.8% in the GEMS group (3/19), and 7.1% (1/14) in the CSCM‐NXC group. (Figure 6).

**FIGURE 7:**
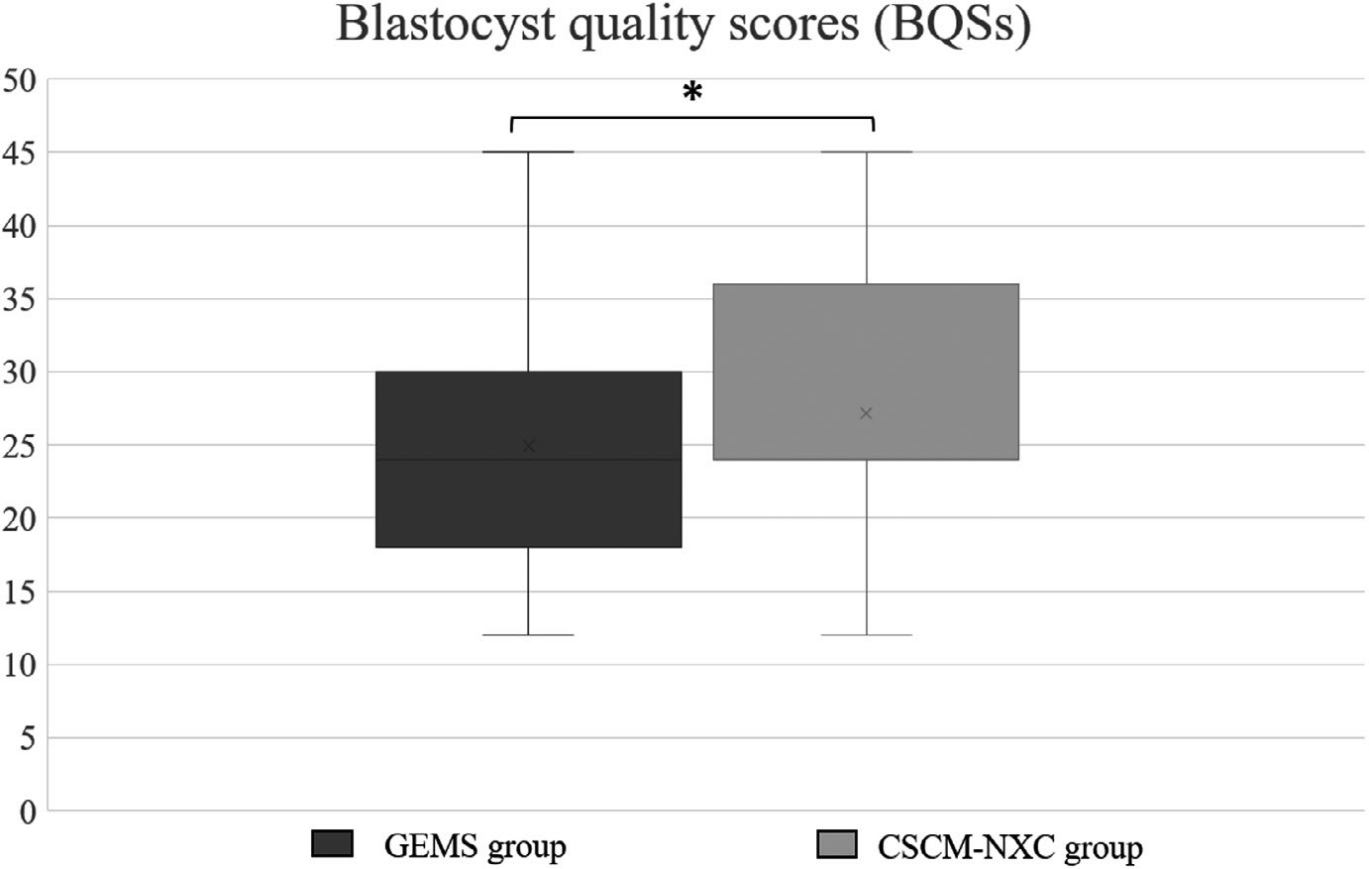
Average value of blastocyst quality scores (BQSs) developed by Khurram et al.^12^ was calculated and compared. The black graph shows GEMS group and the gray graph shows CSCM‐NXC group

The average value of blastocyst quality scores (BQSs) developed by Khurram et al.^12^ was 24.93 in GEMS group and 27.15 in CSCM‐NXC group. The average value of blastocyst quality scores (BQSs) was significantly higher in CSCM‐NXC group compared with GEMS group.(Figure 7).

## 4 DISCUSSION

In this study, it was shown that by using CSCM‐NXC from pre‐culture and insemination, it is possible to obtain good quality embryos with better energy metabolism from the oocyte stage due to the low lactate environment.

Oocytes utilize pyruvate for energy metabolism through oxidative phosphorylation via the TCA circuit and electron‐transfer system, which serves as an important energy source during meiotic maturation.^10^, ^11^

Normally, pyruvate is transferred from the cumulus cells to the oocyte through gap junctions, but pyruvate in the culture medium is directly utilized by the oocyte.^9^

In the absence of cumulus cells or pyruvate, oocyte maturation does not occur in glucose‐containing medium, suggesting that oocytes cannot utilize glucose and must directly utilize cumulus‐derived or medium‐supplied pyruvate to complete meiosis.^9^ From these facts, it is considered that pyruvate is essential for the culture medium. In addition, pyruvate and lactate in the culture medium are believed to be involved in the redox of NAD and NADH in the cytoplasm.

However, an increase in pyruvate may cause ROS generation by oxidative phosphorylation in mitochondria during embryogenesis and inhibit embryonic development.^13^

In addition, it has been reported that pyruvate metabolism is inhibited when lactate in the medium is elevated.^8^, ^14^

Furthermore, it is believed that lactate in the medium at the oocyte/fertilized zygote stage cannot be used for energy metabolism because the fertilized oocyte does not have malate‐aspartate shuttle activity to regenerate NAD in the cytoplasm.^15^

Since NADH is oxidized to NAD in the electron‐transfer system through the mitochondrial inner membrane from the cytoplasm by malate‐aspartate shuttle activity, NAD required for pyruvate conversion is supplied from NAD and lactate available in the TCA circuit. Therefore, pyruvate is considered to be an important energy source for fertilization and early cleavage.

Therefore, reduced lactate concentration rather than an increased pyruvate concentration, from pre‐culture and for insemination increases the uptake and utilization of pyruvate by oocytes.

In addition, pyruvate is produced by the glucose‐based glycolytic system in the cumulus cells and is transported by the oocyte at the gap junction.^16^ It is thought that CSCM‐NXC, a low lactate culture medium, promotes glycolytic metabolism in the cumulus cells as well as in blastocysts, enabling more pyruvate to be delivered to the oocyte.

Energy metabolism before and after fertilization is thought to have a significant effect on culture and clinical outcomes. Energy ATP deficiency at the fertilization stage affects spindle formation and meiotic completion.^17^, ^18^ In addition, ATP deficiency in oocytes leads to dysfunction of the spindle assembly checkpoint (SAC).^19^ Furthermore, abnormalities in oocyte spindle formation have been shown to adversely affect blastocyst formation, implantation, and live birth.^20^

The increase in pyruvate uptake during fertilization reflects the increased energy demand required for the formation of the pronucleus.^21^ Additionally, it has been reported that oocytes with advanced meiosis had higher pyruvate uptake than oocytes arrested at early or mid‐phase meiosis I or II.^22^ It has also been shown that the pyruvate uptake of fertilized embryos, whose development was arrested at the 2‐cell stage, was significantly lower than that of those that reached the blastocyst.^22^

These findings suggest that abnormal energy metabolism at fertilization may cause chromosome segregation errors, leading to split‐sphere apoptosis and embryonic arrest.

In fact, when VerMilyea et al.^23^ compared CSCM‐NX against other companies’ sequential mediums, cultured them to blastocysts, biopsied the trophectoderm, and performed PGT‐A to compare embryo ploidy, the euploid rate of embryos cultured in CSCM‐NX was significantly higher. VerMilyea et al. reported the use of CSCM‐NX in pre‐ICSI pre‐culture, and again, it is possible that oocyte energy metabolism is improved.

These results suggest that the use of CSCM‐NXC, or CSCM‐NX, a low lactate culture medium, in pre‐culture and insemination may increase the efficiency of energy metabolism and lead to a decrease in embryo aneuploidy.

One concern, however, is that CSCM‐NXC has a lower composition of glucose than existing fertilization mediums used in pre‐culture and insemination. Human spermatozoa are thought to produce energy ATP required for the maintenance of flagellar movement, hyperactivation, and capacitation mainly by the glycolytic system using glucose.^24^ Since spermatozoa perform energy metabolism mainly through the glycolytic system, which utilizes glucose, low‐glucose CSCM‐NXC may have a negative effect on spermatozoa in terms of energy metabolism.^25^ However, in the sperm survival test of this study, the sperm motility and sperm concentration did not change when CSCM‐NXC was used as a medium for fertilization compared with GEMS, and it was considered that the number of motile sperm was not reduced. (Table 2).

**TABLE 2:** Sperm survival test

	GEMS group (*n* = 42)	CSCM‐NXC group (*n* = 42)	*p* Value
Age	34.50 ± 8.43		‐
Incubation time (hour)	16.40 ± 2.13		‐
Sperm motility after incubation (%)	75.71	70.15	0.19
Sperm concentration after incubation (×10⁴/ml)	95.20	93.49	0.93

Note

Data are presented as mean ± standard deviation of the mean. After collecting sperms by swim‐up, sperms were incubated in density 50 × 10⁴/ml in GEMS or CSCM‐NX. We performed the calculation of survival data from incubation in 16 ± 2 h and compared those two groups about sperm motility and concentration by *t*‐test, and a significant difference was judged to exist when *p *< 0.05.

It has also been reported that spermatozoa produce NAD by taking up exogenous pyruvate, which promotes energy metabolism by a more glycolytic system.^24^ Therefore, it is possible that the same mechanism that promotes the glycolytic system in the blastocyst by low lactate culture medium can also promote the energy metabolism of sperm. Because of this, it is thought that the number of motile sperm does not decrease and the fertilization rate does not decrease.

In one publication, the fertilization rate did not change in 0.5 mM glucose concentration compared to 5 mM glucose concentration. Also, the number of motile spermatozoa at 4 h of incubation in the sperm survival test did not change, but after 24 h the number of motile spermatozoa was significantly lower in 0.5 mM glucose concentration. Polyspermic fertilization was shown to have occurred only in the group with a glucose concentration of 5 mM.^26^

In addition, it has been reported that glucose concentration is not related to sperm capacitation or acrosome reaction in mice, but oocyte penetration depends on glucose concentration.^18^ In the same article, it was found that one sperm penetrated into an oocyte in culture medium containing no glucose or low glucose concentration (0.5 mM). However, polyspermic fertilization was observed only in the culture medium with high glucose concentration (5.5 mM).^27^ These results suggest that even with a low glucose composition, motile spermatozoa are not drastically reduced, normal fertilization is possible, and the probability of polyspermic fertilization may be reduced.

It has also been reported that immature oocytes are prone to polyspermia at fertilization.^28^, ^29^ This multi‐spermic fertilization is due to the small enclosed oocyte cavity.^30^ Superficial granule release into the enclosed oocyte cavity has been reported to be associated with oocyte maturation.^28^, ^29^ The use of a low lactate culture medium may increase the maturation rate and reduce multiple sperm fertilization because of the good energy metabolism at the oocyte stage.

Although CSCM‐NXC has a glucose concentration sufficient for fertilization as described above and does not inhibit sperm capacitation (hyperactivation), the glucose concentration is lower than that of normal fertilization medium. Consequently, the sperm penetration rate into the oocyte is lower, thus reducing polyspermic fertilization.^27^

As a result of this, CSCM‐NXC, which has a lower glucose and lactate concentration than normal fertilization mediums, can inhibit polyspermic fertilization while maintaining the fertilizing capacity of sperm and is thought to improve the energy metabolism of oocytes, thereby increasing their maturation rate and enabling normal fertilization.

The causes of 1PN are considered to be monogenesis,^31^, ^32^ asynchronous appearance of pronuclei,^32^ fusion of male and female pronuclei, and insufficient nuclear membrane formation in female or male pronuclei.^33^ However, the energy metabolism of sperm is worse than that of oocytes, and asynchrony of pronucleus formation may occur, or the nuclear membrane formation of pronucleus on the sperm side may be defective, but the details are unknown.

The culture chamber used in this study was not a time‐lapse, but a fixed‐point observation, and it is possible that the two pronuclei are only seen overlapping. Although the possibility of monogenicity is considered, there are embryos that have developed into blastocysts from fertilized zygotes of 1PN. (Blastocyst attainment rate of 50% and good quality blastocyst rate of 39% in 1PN‐derived zygotes). Considering this, it is not possible to say with certainty that CSCM‐NXC group has many 1PNs presenting as abnormal fertilization.

In this study, there was a significantly highly expanded blastocysts in the CSCM‐NXC group. Furthermore, the average value of BQSs was significantly higher in the CSCM‐NXC group. At blastocyst stage, ATP is required for Na‐K ATPase to maintain the blastocoel cavity because of the increase in cell number due to the protease enzyme degradation of the clear bodies.^34^ It is thought that the energy metabolism was promoted from the stage of pre‐culture, sperm preparation, and insemination in low lactate medium. Hence, it is possible to think the formation of the blastocoel cavity and the increase in the number of cells by Na‐K ATPase were possible because the energy ATP supply was sufficient from oocyte.

In conclusion, by using CSCM‐NXC, which is a low lactate embryo culture medium, from pre‐culture and for insemination, the energy metabolism efficiency of oocytes and cumulus cells increases, and it becomes possible to supply sufficient energy ATP for fertilization and early cleavage. Additionally, in using low lactate CSCM‐NXC, in total, including pre‐culture and for insemination through blastocyst culture, efficient energy metabolism can continue to promote good embryo development and increase the number of embryos that can be transferred.

## CONFLICT OF INTEREST

The authors declare no conflicts of interest associated with this manuscript.

## ETHICAL APPROVAL

All procedures followed were in accordance with the ethical standards of the responsible committee on human experimentation (institutional and national) and with the Helsinki Declaration of 1964 and its later amendments. This article does not contain any studies with animal subjects performed by any of the authors. The protocol for the research project including human subjects has been approved by a suitably constituted Ethics Committee.

## INFORMED CONSENT

Informed consent was obtained from all patients for being included in the study.
